# Classification of Seasonal Allergic Rhinitis Patients by Prospective Patient‐Recorded Symptoms: The @IT.2020 Project

**DOI:** 10.1111/all.70447

**Published:** 2026-09-02

**Authors:** Stephanie Dramburg, Camilo J. Hernandez Toro, Ulrike Grittner, Ekaterina Potapova, Alessandro Travaglini, Salvatore Tripodi, Stefania Arasi, Simone Pelosi, Francesco Monnati, Aydan Acar Şahin, Xenophon Aggelidis, Andrea Barbalace, Anne Bourgoin, Blerina Bregu, Maria Antonia Brighetti, Elsa Caeiro, Sule Caglayan Sozmen, Lucia Caminiti, Denis Charpin, Mariana Couto, Luís Delgado, Andrea Di Rienzo Businco, Claire Dimier, Maria V. Dimou, João A. Fonseca, Ozlem Goksel, Dolores Hernandez, Tara Maria Hoffmann, Dah Tay Jang, Fusun Kalpaklioglu, Blerta Lame, Ruth Llusar, Michael P. Makris, Angel Mazon, Eris Mesonjesi, Antonio Nieto, Ayse Bilge Öztürk, Laurie Pahus, Giovanni Battista Pajno, Ilenia Panasiti, Nikolaos G. Papadopoulos, Elisabetta Pellegrini, Ana Margarida Pereira, Mariana Pereira, Munevver Pinar, Alfred Priftanji, Fotis Psarros, Cansin Sackesen, Ifigenia Sfika, Javier Suarez, Michel Thibaudon, Ulas Uguz, Valentine Verdier, Valeria Villella, Paraskevi Xepapadaki, Duygu Yazici, Paolo M. Matricardi

**Affiliations:** ^1^ Department of Pediatric Respiratory Care, Immunology and Intensive Care Medicine Charité – Universitätsmedizin Berlin, Corporate Member of Freie Universität Berlin and Humboldt‐Universität zu Berlin Berlin Germany; ^2^ Institute of Biometry and Clinical Epidemiology, Charité ‐ Universitätsmedizin Berlin Berlin Germany; ^3^ Department of Biology Tor Vergata University Rome Italy; ^4^ Italian Aerobiology Monitoring Network ‐ Italian Aerobiology Association Rome Italy; ^5^ Pediatric Allergy Unit Sandro Pertini Hospital Rome Italy; ^6^ Allergolology Service Policlinico Casilino Rome Italy; ^7^ Pediatric Allergology Unit, Department of Pediatric Medicine Bambino Gesù Children's Research Hospital (IRCCS) Rome Italy; ^8^ TPS Production Srl Rome Italy; ^9^ INFN & Department of Physics Tor Vergata University of Rome Rome Italy; ^10^ Fraunhofer Institute for Translational Medicine and Pharmacology ITMP, Immunology and Allergology Berlin Germany; ^11^ Department of Biology, Faculty of Science Ankara University Ankara Turkey; ^12^ Allergy Unit, 2nd Department of Dermatology and Venereology, National and Kapodistrian University of Athens University Hospital “Attikon” Athens Greece; ^13^ Department of Pediatrics‐Allergy Unit University of Messina Messina Italy; ^14^ Department of Pneumonology and Allergy La Timone Hospital, APHM, Aix‐Marseille University Marseille France; ^15^ Department of Allergology and Clinical Immunology UHC Mother Teresa, Medical University Tirana Tirana Albania; ^16^ MED Mediterranean Institute for Agriculture, Environment and Development & CHANGE Global Change and Sustainability Institute, Institute for Advanced Studies and Research Universidade de Évora Évora Portugal; ^17^ Portuguese Society of Allergology and Clinical Immunology Lisbon Portugal; ^18^ Medicana International Izmir Hospital Izmir Turkey; ^19^ Immunoallergology Hospital CUF Trindade Porto Portugal; ^20^ Basic and Clinical Immunology Unit, Department of Pathology, Faculty of Medicine University of Porto Porto Portugal; ^21^ RISE‐Health Faculty of Medicine of the University of Porto Porto Portugal; ^22^ Allergy Unit Instituto & Hospital CUF Porto Porto Portugal; ^23^ Allergy Department, 2nd Pediatric Clinic, Athens General Children's Hospital “P&A Kyriakou” University of Athens Athens Greece; ^24^ Department of Pulmonary Medicine, Division of Immunology, Allergy and Asthma. Faculty of Medicine Ege University Izmir Turkey; ^25^ Department of Allergy Health Research Institute Hospital La Fe Valencia Spain; ^26^ Pediatric Allergy and Pneumology Unit Children's Hospital La Fe; Health Research Institute La Fe Valencia Spain; ^27^ Department of Immunology and Allergic Diseases Kırıkkale University School of Medicine Kırıkkale Turkey; ^28^ Department of Pulmonary Medicine, Division of Allergy and Immunology Medeniyet University, School of Medicine Istanbul Turkey; ^29^ Aix Marseille Univ, APHM, INSERM CIC 1409, INSERM U1263, INRA 1260 (C2VN) Marseille France; ^30^ Division of Infection, Immunity & Respiratory Medicine, Royal Manchester Children's Hospital University of Manchester Manchester UK; ^31^ Department of Reggio Calabria ARPA‐ Regional Agency for Environmental Protection Calabria Italy; ^32^ Allergy Department Athens Naval Hospital Athens Greece; ^33^ Division of Pediatric Allergy Koç University School of Medicine Istanbul Turkey; ^34^ Department of Biology of Organisms and Systems, Area of Botany University of Oviedo Oviedo Spain; ^35^ Réseau National de Surveillance Aérobiologique Brussieu France; ^36^ Department of Biology, Faculty of Science Ege University Izmir Turkey; ^37^ Allergy Department, 2nd Pediatric Clinic National and Kapodistrian University of Athens Athens Greece; ^38^ Cellular and Molecular Medicine, KUTTAM, Graduate School of Health Sciences Koç University Istanbul Turkey; ^39^ Swiss Institute of Allergy and Asthma Research (SIAF) University of Zurich Davos Switzerland; ^40^ Institute of Allergology, Charité – Universitätsmedizin Berlin Corporate Member of Freie Universität Berlin and Humboldt‐Universität zu Berlin Berlin Germany

**Keywords:** cluster analysis, e‐Diary, mHealth, patient‐reported outcomes, seasonal allergic rhinitis classification

## Abstract

**Background:**

Seasonal allergic rhinitis (SAR) has been so far classified according to frequency and severity by retrospective information based on the ARIA (Allergic Rhinitis and its Impact on Asthma) classification. Electronic symptom diaries (e‐Diaries) are feasible tools to collect prospective patient‐reported data.

**Objective:**

To test the consistency between prospectively recorded symptoms and retrospective ARIA classification and to generate a new SAR classification based on prospective e‐Diary data.

**Methods:**

Within the @IT.2020 multicentre observational study, we recruited 815 children and adults with SAR in nine study centres from Mediterranean countries. Participants recorded daily organ‐specific (Rhinoconjunctivitis Total Symptom Score; RTSS) and general symptoms (visual analogue scale, VAS) plus quality‐of‐life measures via an e‐Diary app. Retrospective clinical questionnaires were performed at the beginning (T0) and end (T1) of the pollen season. Unsupervised clustering analyses around medoids were implemented to classify SAR by prospective patient‐reported data.

**Results:**

One third (56/164, 34.1%) and almost half (173/366, 47.3%) of the patients retrospectively over‐ or underestimated the frequency of their symptoms, respectively. A moderate correlation was found between SAR severity reported at T1 and prospectively recorded symptom severity. An unsupervised clustering approach was able to identify distinct groups (mild, moderate, severe cluster) of patients based on the prospective severity of organ‐specific (RTSS) and overall symptoms (VAS), as well as quality of life criteria.

**Conclusions:**

A significant recall bias regarding frequency and severity of symptoms emerged by comparing the retrospective ARIA classification with prospectively recorded data by an e‐Diary. A prospective approach to SAR classification, inspired by the retrospective ARIA classification, may facilitate a more precise evaluation of the patient for improved disease management.

## Graphical abstract

Daily prospective symptom reports from participants in this multicentre observational study were compared to retrospective (last 12 months) reports based on ARIA clinical questionnaire. Retrospective reports substantially underestimated, and to a lesser extent overestimated, symptom frequency compared to e‐Diary prospective reports. Since retrospective severity classes poorly matched prospective symptom severity averages, a new severity classification based on clustering around medoids of the prospective data collected through the e‐Diary is proposed. AIT, allergen immunotherapy; ARIA, allergic rhinitis and its impact on asthma; ATH, Athens; IST, Istanbul; IZM, Izmir; MAR, Marseille; MES, Messina; POR, Portugal; ROM, Rome; TIR, Tirana; VAL, Valencia.

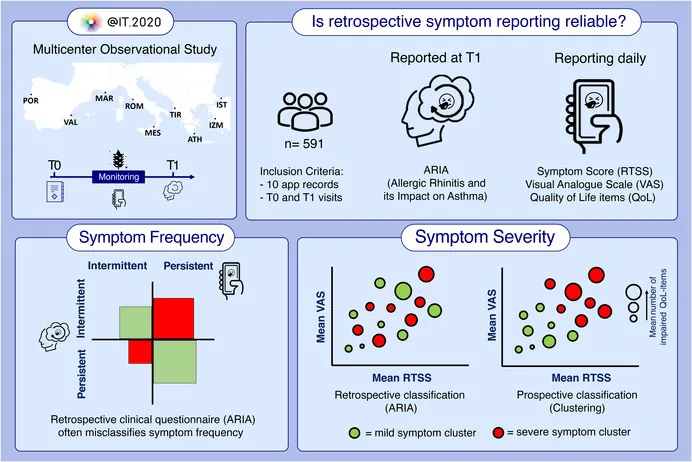

**Abbreviations**
AIT: allergen immunotherapy
ARIA: Allergic Rhinitis and its Impact on Asthma
ATH: Athens
IST: Istanbul
IZM: Izmir
MAR: Marseille
MES: Messina
POR: Portugal
QoL: quality of life
ROM: Rome
RTSS: rhinoconjunctivitis total symptom score
SAR: seasonal allergic rhinitis
SD: standard deviation
SMD: Standardised Mean Difference
SPT: skin prick test
TIR: Tirana
VAL: Valencia
VAS: visual analogue score

## 1 Introduction

The clinical picture of seasonal allergic rhinoconjunctivitis (SAR) due to airborne pollen grains and fungal spores is heterogeneous, as phenotypes change geographically with allergen exposure [1]. This demanding combination presents a challenge when it comes to finding the optimal treatment strategy for each patient [2] and environmental area [1]. Whether this implies patient education [3], symptomatic treatment [4] or the only causal treatment, allergen‐specific immunotherapy (AIT) [4], depends largely on the severity and frequency of symptoms affecting the nose, eyes and lungs [5].

A tailored prescription of AIT requires both a precise identification of the eliciting allergen and a careful evaluation of the patient's quality of life achieved with drug treatment only [5]. This evaluation is usually done retrospectively with a short questionnaire, very often the Allergic Rhinitis and its Impact on Asthma (ARIA) classification questions [6].

However, retrospective clinical assessments carry the risk of recall bias [7], while prospective clinical diaries are more sensitive and precise in catching day‐by‐day variations of both airborne pollen exposure and patients' allergy symptoms [8]. Several smartphone applications have been created to enable prospective and continuous symptom monitoring in SAR patients [9, 10, 11, 12]. The wide use of the app MASK‐Air led to large real‐life data sets of more than 63,000 users in 30 countries [13] This valuable information led to the identification of new allergic rhinitis, rhinoconjunctivitis and asthma phenotypes in real life [14], a better understanding of medication adherence and disease control [15], and the development and validation of different disease severity scores [13].

However, most users who spontaneously download an e‐Diary tend to use the app for only a few days to weeks [16, 17, 18], which makes the interpretation of the course of disease difficult. By contrast, in the @IT.2020 pilot and multicentre studies, about 80% of the participants recorded their symptom data for 90 days or longer [19, 20]. This valuable performance was achieved in a so‐called ‘blended approach’, combining remote monitoring with previous patient education by a healthcare professional with independent monitoring and a joint interpretation of the collected data [20].

In 91 Italian participants of the @IT.2020 pilot study, we compared retrospective (Allergic Rhinitis and its Impact on Asthma (ARIA) questionnaire) with prospective (e‐Diary) information on patients' symptoms, medication and quality of life [21]. Interestingly, we found that SAR patients overestimate the frequency of their symptoms when interrogated after the end of the pollen season, and also severity measures correlated only partially between prospective and retrospective data [20]. This observation led to the hypothesis that AIT prescription would be more precise if based on an e‐Diary based examination of the SAR patients [7]. We aimed this study at testing whether the partial concordance between retrospective and prospective symptom reporting holds true in diverse geographical and cultural scenarios, characterised by different airborne pollen exposure, sensitisation profiles, and disease burden. To this end, we repeated the comparative study setting of the pilot study in the multicentre phase of the @IT‐2020 project, recruiting SAR patients in nine highly heterogeneous Southern European urban areas [1]. To expand the previous analyses, we further applied an unsupervised clustering approach to stratify patients based on prospectively collected symptom severity.

## 2 Materials and Methods

### 2.1 Study Population

The @IT.2020 Observational Longitudinal Multicentre Clinical Study was conducted in 2017 and 2018 to evaluate the impact of molecular IgE testing and digital symptom recording on the diagnosis of SAR in Southern Europe. Paediatric and adult patients diagnosed with SAR were recruited in nine urban study centres located in seven Southern European countries (Porto, Portugal; Valencia, Spain; Marseille, France; Rome and Messina, Italy; Tirana, Albania; Athens, Greece; Istanbul and Izmir, Turkey). In addition to a SAR diagnosis, inclusion criteria were: (i) age 10–60 years; (ii) a good understanding of the national language or one of the languages offered in the mobile study application (Allergymonitor, TPS software production, Rome, Italy); (iii) availability of a smartphone; (iv) written informed consent. Exclusion criteria were: (i) prior AIT for pollen allergies; (ii) any severe chronic disease; (iii) living more than 30 km away from the local pollen trap. Patients with at least 10 (not necessarily consecutive) days with symptom self‐reports in the study app were included in the current analyses. More detailed information on the study protocol has been published previously [1, 21, 22].

### 2.2 Study Design

Recruited patients underwent a visit (T0) dedicated to the blood collection, skin prick testing (SPT) with a comprehensive set of seasonal and perennial aeroallergens, and compilation of clinical questionnaires in an interview with the study doctor. Based on the individual clinical history and SPT results, every participant received an individual monitoring period matching the expected airborne pollen/spore levels of the relevant source(s). During this time, symptoms were to be recorded daily via the e‐diary app Allergymonitor. In parallel, airborne pollen and spore concentrations were monitored at the local aerobiological centre. Details on the aerobiological monitoring as well as clinical symptoms and allergen‐specific IgE profiles have been published previously [1, 20, 21]. After the individually relevant pollen season, participants attended a final study visit (T1), during which the retrospective questionnaires (ARIA) regarding that past season were filled.

### 2.3 Questionnaires and Scores

#### 2.3.1 Retrospective Evaluation of Symptom Severity and Frequency

The ARIA classification is a worldwide used tool to retrospectively assess the severity and frequency of allergic rhinitis symptoms [6]. It includes four questions regarding disease‐related quality of life, namely whether the symptoms affect a patient's (i) sleep, (ii) leisure activities/sport, (iii) school/work, and (iv) whether symptoms are perceived as particularly troublesome. In a modified ARIA score, severity is judged as mild when none of these questions are answered with ‘yes’, while 1–3 or all 4 affected items lead to a classification in moderate and severe, respectively [23]. SAR frequency is ‘intermittent’ if symptoms occur on less than 4 days per week or during less than 4 consecutive weeks, or ‘persistent’ when symptoms occur during 4 or more days per week and for more than 4 consecutive weeks [6]. We obtained baseline ARIA classification of SAR affected participants at T0 for the 12 months preceding the study. We also obtained an ARIA classification at T1, referred to the pollen season 2018 only. The ARIA classification obtained at T1 has been compared to the SAR classification based on the patient's e‐Diary data collected during the same pollen season (2018).

#### 2.3.2 Prospective symptom severity

The e‐Diary AllergyMonitor offers a daily assessment of organ‐specific questions for symptoms of the eyes (itching tearing/reddening) and nose (sneezing, itch, rhinorrhea, blocking). Based on this information, the Rhinoconjunctivitis Total Symptom Score (RTSS) was calculated (range 0–12) [24]. Patients also fill questions concerning lung symptoms (night/day cough, dyspnoea, need for doctor's assistance), allowing an assessment in accordance with the GINA guidelines [8]. Finally, participants were asked to indicate their overall symptom severity via a visual analogue scale (VAS) (‘How much were you affected by your allergy symptoms?’, 0 = not at all → 10 = very much).

#### 2.3.3 Prospective Quality of Life Assessment

The daily questionnaire in the AllergyMonitor e‐Diary also included all four questions on disease‐related quality of life from the ARIA assessment (sleep, leisure activities, school/work, troublesome symptoms).

### 2.4 Statistical Analyses

Categorical data were summarised as absolute and relative frequencies, and continuous data was summarised as mean and standard deviation or as median, first and third quartiles. To provide a standardised effect size of differences in baseline and app record descriptors, the mean Standardised Mean Difference (SMD) of pairwise comparisons between study centres, ARIA severity groups at T1, and participants included and excluded from the analyses was calculated. SMD cut‐off points of 0.20, 0.50 and 0.80 were considered to represent a small, moderate, and large effect, respectively [25]. For more details on the statistical methods for prospective data analysis, see the Online Repository.

Missing daily e‐Diary entries were imputed by linear interpolation for a maximum of 3 consecutive missing days with the assumption of Missing at Random. To identify groups of patients based on the averages of the prospectively recorded scores, unsupervised clustering analyses around medoids (a more robust version of k‐means) were performed. The average from each score was taken within the days belonging to the fragmented season(s) (i.e., taking only days with relevant pollen exposure into account by applying season definitions according to Hoffmann et al. [22]) relevant to each patient. With the intention to compare with the three severity classes defined by the ARIA classification (mild, moderate, severe), the main analysis was performed with three clusters. As a sensitivity analysis, the three‐group clustering described previously was repeated with a subset of patients with high‐quality data (more details in the Online Repository). The clustering was then repeated using only the patients belonging to each of the study centres. The separating lines between clusters were estimated through a grid search in the RTSS‐VAS space for the clustering with all patients as well as for the clustering independently trained with data from each study centre.

As an attempt to determine the importance of each of the scores on the determination of the clusters, the clustering procedure (*k* = 3) was repeated using all possible subsets of the score averages for model training and the results of these clustering analyses were compared via contingency tables to the clustering performed with all three mean scores. Average balanced accuracy, sensitivity, and specificity across all three classes were estimated for each comparison.

As an attempt to evaluate the generalizability of the decision boundaries found via grid search on the RTSS‐VAS space, a bootstrap estimate of these boundaries was performed following the same approach described before. A total of 1000 bootstrap samples were drawn from the set of all patients, and the mean boundary, as well as its 95% bootstrapped confidence interval were estimated. Additionally, bootstrap estimates of cluster stability (i.e., Jaccard Index) were also estimated.

### 2.5 Statistical Software

All statistical analyses were conducted using R (version 4.2.2) (http://www.r‐project.org/). The ‘tableone’ (version 0.13.2) [26] library was used to generate summary statistics and estimate mean SMD, ‘lme4’ (version 1.1.34) [27] to fit linear and generalised linear mixed‐effects models, ‘sjPlot’ (version 2.8.15) [28] to create mixed‐effects models summary tables, ‘cluster’ (version 2.1.4) [29] and ‘fpc’ (version 2.2–13) [30] to perform clustering analyses, ‘zoo’ (version 1.8.12) [31] for moving window aggregations and smoothing, ‘caret’ (version 6.0.94) [32] to estimate metrics from confusion matrices, and ‘ggplot2’ (version 3.5.1) [33] and ‘ggpubr’ (version 0.6.0) [34] for plotting.

## 3 Results

### 3.1 Study Population

Of the included 815 pollen allergic patients (348 children, 467 adults), 591 had at least 10 e‐Diary recordings during the relevant pollen season and completed the T1 visit. The mean age of included patients was 26.2 years (SD 13.7) and 45.5% (*n* = 269) of the participants were female. The most frequent allergic comorbidities were asthma, urticaria and atopic dermatitis with no large differences between study centres (SMD < 0.5 for all comorbidities). More details on the clinical phenotypes and sensitization profiles of participants have been published elsewhere [1]. A persistent, moderate SAR phenotype was the most frequent at T0 (Table 1) and a moderate but intermittent phenotype at T1, that is after the monitoring period (pollen season 2018) (Table 1), independently from age or gender. A comparison of baseline characteristics between in‐ and excluded patients yielded no large differences (Table e1).

**TABLE 1:** Baseline characteristics of the study population.

	ATH (*n* = 77)	IST (*n* = 53)	IZM (*n* = 73)	MAR (*n* = 71)	MES (*n* = 49)	POR (*n* = 77)	ROM (*n* = 79)	TIR (*n* = 86)	VAL (*n* = 26)	Overall (*n* = 591)	SMD
Female gender; *n* (%)	25 (32.5)	31 (58.5)	38 (52.1)	36 (50.7)	19 (38.8)	33 (42.9)	36 (45.6)	39 (45.4)	12 (46.2)	269 (45.6)	0.18
Age											
Mean (SD)	23.19 (12.5)	26.49 (13.0)	23.96 (14.7)	39.54 (10.1)	23.47 (12.4)	25.09 (13.0)	26.73 (16.0)	26.14 (10.3)	12.42 (1.6)	26.25 (13.7)	0.70
Comorbidities; *n* (%)											
Oral allergy syndrome	6 (7.8)	0 (0)	10 (13.7)	6 (8.5)	12 (24.5)	12 (15.6)	25 (31.7)	14 (16.3)	0 (0)	85 (14.4)	0.40
Anaphylaxis	3 (3.9)	0 (0)	5 (6.9)	2 (2.8)	3 (6.1)	7 (9.1)	3 (3.8)	9 (10.5)	0 (0)	32 (5.4)	0.22
Urticaria	10 (13.0)	2 (3.8)	13 (17.8)	22 (31.0)	16 (32.7)	9 (11.7)	29 (36.7)	32 (37.2)	5 (19.2)	138 (23.4)	0.37
Atopic dermatitis	11 (14.3)	6 (11.4)	7 (9.6)	24 (33.8)	11 (22.5)	21 (27.3)	23 (29.1)	21 (24.4)	12 (46.2)	136 (23.0)	0.33
Food allergy	8 (10.39)	4 (7.6)	3 (4.1)	11 (15.5)	3 (6.1)	11 (14.3)	14 (17.7)	13 (15.1)	5 (19.2)	72 (12.2)	0.21
Asthma (ever)	39 (50.65)	16 (30.2)	44 (60.3)	32 (45.1)	22 (44.9)	16 (20.8)	35 (44.3)	13 (15.1)	8 (30.8)	225 (38.1)	0.38
Other atopic disease	5 (6.49)	1 (1.9)	2 (2.7)	1 (1.4)	1 (2.04)	0 (0)	5 (6.3)	2 (2.3)	0 (0)	17 (2.9)	0.17
Number of pollen allergies; *n* (%)											
1	5 (6.49)	19 (35.9)	25 (34.3)	24 (33.8)	17 (34.7)	29 (37.7)	6 (7.6)	7 (8.1)	10 (38.5)	142 (24.1)	0.81
2	14 (18.2)	15 (28.3)	19 (26.0)	21 (29.6)	20 (40.8)	24 (31.2)	11 (13.9)	20 (23.3)	10 (38.5)	154 (26.1)	
3	21 (27.3)	9 (17.0)	9 (12.3)	15 (21.1)	9 (18.4)	8 (10.4)	15 (19.0)	19 (22.1)	4 (15.4)	109 (18.4)	
4	16 (20.8)	6 (11.3)	9 (12.3)	10 (14.1)	1 (2.0)	4 (5.2)	17 (21.5)	8 (9.3)	1 (3.9)	72 (12.2)	
5	10 (13.0)	3 (5.7)	4 (5.5)	1 (1.4)	2 (4.1)	5 (6.5)	19 (24.1)	11 (12.8)	1 (3.9)	56 (9.5)	
> 5	11 (14.3)	1 (1.9)	7 (9.6)	0 (0)	0 (0)	7 (9.1)	11 (13.9)	21 (24.4)	0 (0)	58 (9.8)	
Positive SPT to perennial aeroallergens^a^; *n* (%)	44 (57.1)	21 (39.6)	26 (35.6)	28 (39.4)	33 (67.4)	43 (55.8)	53 (67.1)	51 (59.3)	12 (46.2)	311 (52.6)	0.30
ARIA at T0; *n* (%)											
Mild intermittent	2 (2.6)	2 (3.8)	4 (5.5)	1 (1.4)	4 (8.2)	0 (0)	0 (0)	6 (7.0)	5 (19.2)	24 (4.1)	0.87
Mild persistent	2 (2.6)	9 (17.0)	4 (5.5)	2 (2.8)	7 (14.3)	2 (2.6)	6 (7.6)	0 (0)	10 (38.5)	42 (7.1)	
Moderate intermittent	8 (10.4)	6 (11.3)	14 (19.2)	2 (2.8)	11 (22.5)	16 (20.8)	2 (2.5)	7 (8.1)	2 (7.7)	68 (11.5)	
Moderate persistent	40 (52.0)	27 (50.9)	23 (31.5)	26 (36.6)	18 (36.7)	31 (40.3)	57 (72.2)	35 (40.7)	8 (30.8)	265 (44.8)	
Severe intermittent	3 (3.9)	1 (1.9)	3 (4.1)	7 (9.9)	2 (4.1)	5 (6.5)	0 (0)	3 (3.5)	0 (0)	24 (4.1)	
Severe persistent	22 (28.6)	8 (15.1)	25 (34.3)	33 (46.5)	7 (14.3)	23 (29.9)	14 (17.7)	35 (40.7)	1 (3.9)	168 (28.4)	
ARIA at T1; *n* (%)											
Mild intermittent	19 (24.7)	20 (37.7)	14 (19.2)	6 (8.5)	10 (20.4)	5 (6.5)	13 (16.5)	15 (17.4)	2 (7.7)	104 (17.6)	0.80
Mild persistent	8 (10.4)	6 (11.3)	10 (13.7)	3 (4.2)	1 (2.1)	0 (0)	6 (7.6)	1 (1.2)	3 (11.5)	38 (6.4)	
Moderate intermittent	21 (27.3)	11 (20.8)	13 (17.8)	13 (18.3)	14 (28.6)	44 (57.1)	22 (27.9)	23 (26.7)	10 (38.5)	171 (28.9)	
Moderate persistent	22 (28.6)	6 (11.3)	17 (23.3)	27 (38.0)	18 (36.7)	10 (13.0)	34 (43.1)	23 (26.7)	10 (38.5)	167 (28.3)	
Severe intermittent	2 (2.6)	9 (17.0)	5 (6.9)	3 (4.2)	2 (4.1)	10 (13.0)	1 (1.3)	9 (10.5)	1 (3.9)	42 (7.1)	
Severe persistent	5 (6.5)	1 (1.9)	14 (19.2)	19 (26.8)	4 (8.2)	8 (10.4)	3 (3.8)	15 (17.4)	0 (0)	69 (11.7)	

^a^ House dust mite, cat, dog.

At T1, asthma was most frequent among patients with severe (50/111, 45.1%), compared to those with moderate (118/338, 34.9%) or mild (57/142, 40.1%) SAR (Table e2). SAR severity was neither related to the number of different pollen allergies nor to sensitisation to perennial aeroallergens (house dust mites, cat, and dog). Finally, retrospective SAR classification was different at T0 compared to T1 in 417/591 patients (70.6%). Most remarkably, a large proportion 112/142 (78.9%) of the mildly affected group at T1 had moderate to severe SAR at T0 (Table e3).

### 3.2 Prospectively Recorded Data and Their Relationship With Retrospective Evaluations

#### 3.2.1 Prospective Versus Retrospective Evaluation of Disease Severity

Mean organ‐specific disease severity (RTSS) (Figure 1), general disease severity (VAS) and impact on quality of life (QoL) (Figure e1) across all patients were assessed in all centres covering the most relevant pollen seasons. Figure 1 shows the respective average symptom severity score trajectories and their standard deviations, by retrospectively assessed symptom severity group (ARIA during T1: mild, moderate, severe). As expected, prospective trajectories show a higher, intermediate, and lower disease severity among patients retrospectively classified as ‘severe’, ‘moderate’ or ‘mild’ at T1. As expected, considering that the ARIA severity classification is based on questions regarding quality‐of‐life, the power of a prospective recording in discriminating SAR severity categories was the highest when the QoL questions were considered, especially in ATH, IST, IZM, MAR and ROM (Figure 1c). To better analyse this relevant aspect, we examined differences in the means of average per‐patient scores by study centre (Figure e2) and by retrospective (ARIA, T1) severity classification using the SMD (Table e4). QoL questions (SMD 0.62) displayed the highest discriminant ability for T1 ARIA classification with a mean score of 0.92 (SD 1.05) in the severe group versus 0.42 (SD 0.71) and 0.20 (SD 0.44) in the moderate and mild group, respectively (Figure 2, Table e2).

**FIGURE 1:**
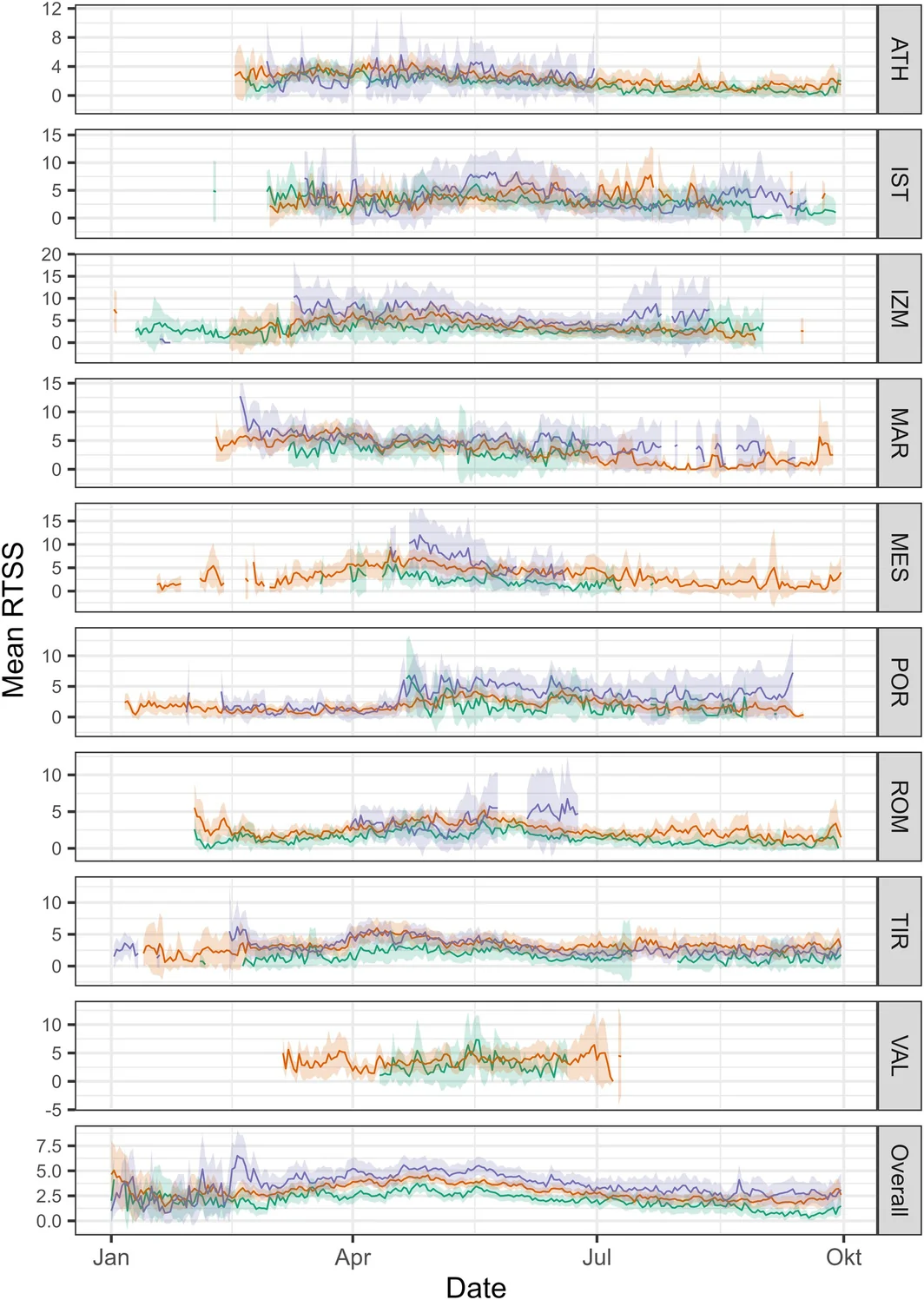
Prospectively recorded symptom trajectory (rhinoconjunctivitis total symptom score, RTSS) by retrospectively determined severity class (ARIA) and study centre; mean population data plus 95% confidence interval are shown. Plots are only shown for dates in which ≥ 3 patients reported the respective symptom severity. Details on group sizes are shown in Table 1. ATH: Athens (*n* = 77), IST: Istanbul (*n* = 53), IZM: Izmir (*n* = 73), MAR: Marseille (*n* = 71), MES: Messina (*n* = 49), POR: Portugal (*n* = 76), ROM: Rome (*n* = 79), TIR: Tirana (*n* = 86), VAL: Valencia (*n* = 26).

**FIGURE 2:**
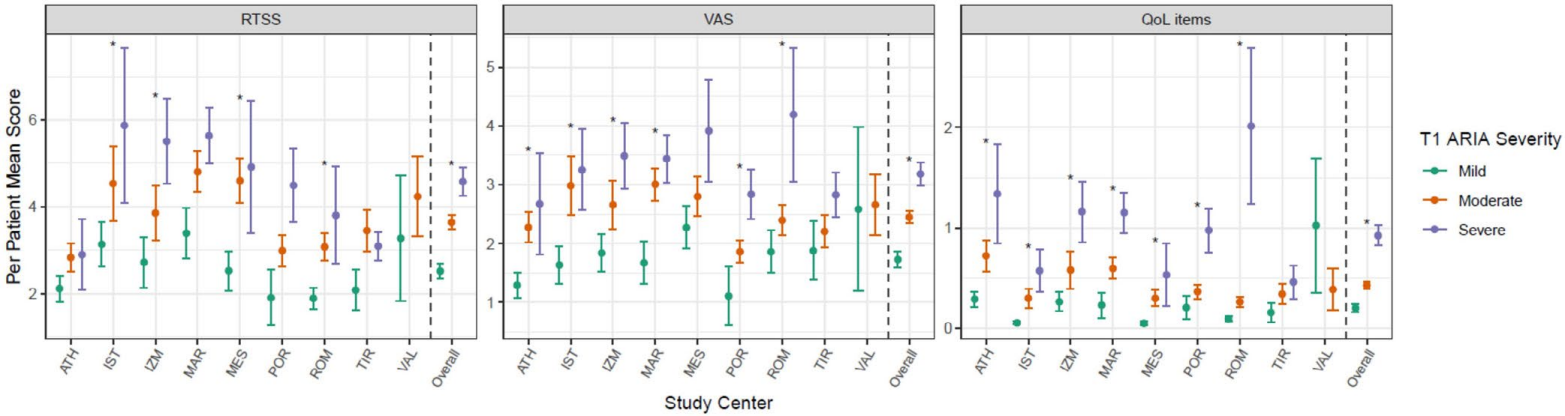
Prospective mean and standard error of disease severity by study centre and retrospective severity group (ARIA at T1); RTSS: Rhinoconjunctivitis Total Symptom Score (0–18), VAS: Visual Analogue Scale (0–10) of overall symptoms, QOL items: Sum of sleep impairment, work/school productivity impairment, affection of leisure activities, and perception of troublesome symptoms (0–4). Group sizes can be found in Table 1. ATH: Athens (*n* = 77), IST: Istanbul (*n* = 53), IZM: Izmir (*n* = 73), MAR: Marseille (*n* = 71), MES: Messina (*n* = 49), POR: Portugal (*n* = 76), ROM: Rome (*n* = 79), TIR: Tirana (*n* = 86), VAL: Valencia (*n* = 25, severe group consisted of only 1 patient; this data was removed).

As the impairment of quality of life is the parameter used to distinguish ARIA severity groups, we also analysed the proportion of days during which patients indicated suffering from impairment in one or more of the quality‐of‐life domains. In every domain (troublesome symptoms, impairment of daily activities/leisure, sleep disturbance and impairment of school/work activities), patients of the severe group according to T1 ARIA reported the highest proportion of days as affected, followed by the moderate and mild groups (Figure 3). Incidence Rate Ratios estimated using Poisson regression models to analyse the association between the number of days with reports of affected QoL items and T1 ARIA severity showed similar results. A detailed description of the models is available in the Supporting Information: Methods.

**FIGURE 3:**
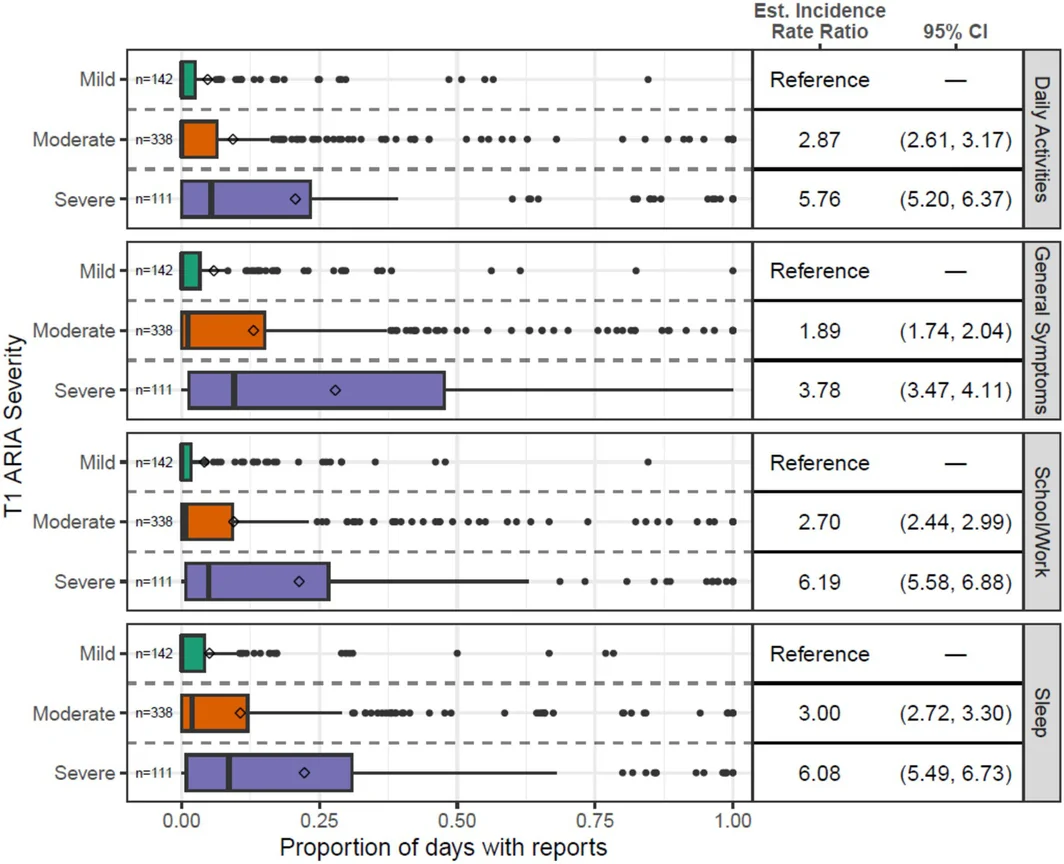
Proportion of days with reports of affected quality of life and estimated Incidence Rate Ratios with 95% CI from Poisson regressions of the number of days in which each QoL item was reported by each patient, with total number of days with reports from each patient as offset, T1 ARIA severity as independent variable, and a random intercept by study centre. From top to bottom: affected daily activities/leisure, symptoms perceived as particularly troublesome, impairment of school/work activity, and sleep impairment. Diamond in boxplots shows the mean proportion of days with reports from each QoL item by T1 ARIA severity.

An analysis by study centre revealed similar results across all centres with few variations. When analysing the prospective mean disease severity by ARIA T1 severity, per patient mean scores were associated with the severity group across all study centres but Valencia, where only one patient was classified as severely affected at T1 (Figure 2). Additionally, patients in Messina seem to be mainly troubled by sleep impairment, while participants in other centres show a more homogeneous distribution of QoL impairment across domains (e.g., Rome, Marseille) (Figure e3).

#### 3.2.2 Prospective Versus Retrospective Evaluation of Symptom Frequency

When comparing the recorded frequency of symptoms during the monitoring period to the retrospective estimates of patients, a clear discrepancy becomes apparent. As recall bias will most likely hamper the retrospective evaluation, we decided to consider the prospectively reported data as point of reference, which resulted in a balanced accuracy of 0.59 for the retrospective data. In detail, 56 of the 164 (34.1%) patients who reported symptoms for less than 4 days/week and during less than 4 consecutive weeks in the e‐Diary (intermittent phenotype according to ARIA classification) had retrospectively assessed their symptoms as persistent. On the other hand, 173/366 (47.3%) patients had retrospectively underestimated their symptom frequency as being intermittent (Table 2).

**TABLE 2:** Sensitivity, specificity and balanced accuracy of retrospective versus prospective symptom data (considering prospective frequency as point of reference).

		Frequency e‐Diary		
		Intermittent	Persistent	Total
Frequency T1 ARIA	Intermittent	108	173	281
	Persistent	56	193	249
	Total	164	366	530

*Note:* Comparison of prospective (frequency from e‐Diary) and retrospective (frequency from ARIA classification at T1) SAR symptom frequency. Prospective frequency was considered as reference and "Persistent" as the positive class. Balanced accuracy = 0.59, Sensitivity = 0.53, Specificity = 0.66.

### 3.3 Prospective Symptom Severity Clustering Identifies Distinct Groups

A graphical illustration of the prospectively recorded symptom data in a bubble plot (Figure 4a) shows a heterogeneous overlap of patients from all retrospective ARIA severity groups (red: severe, blue: moderate, green: mild) in their average prospective self‐reported SAR symptom severities (i.e., VAS and RTSS). As quality‐of‐life items are the decisive measures for disease severity in the ARIA classification, we performed unsupervised clustering around medoids based exclusively on the QoL items [Figure 4b]. This approach was also insufficient to clearly differentiate groups of patients in terms of prospective self‐reported SAR symptom severity. A second unsupervised clustering approach including the per‐patient average prospective VAS and RTSS, however, was able to identify groups of patients according to the prospective severity of organ‐specific (RTSS), overall (VAS) symptoms, and the number of QoL items affected daily (Figure 4c). These clusters were very similar to those found in the sensitivity analysis with patients with high‐quality data (Table e6).

**FIGURE 4:**
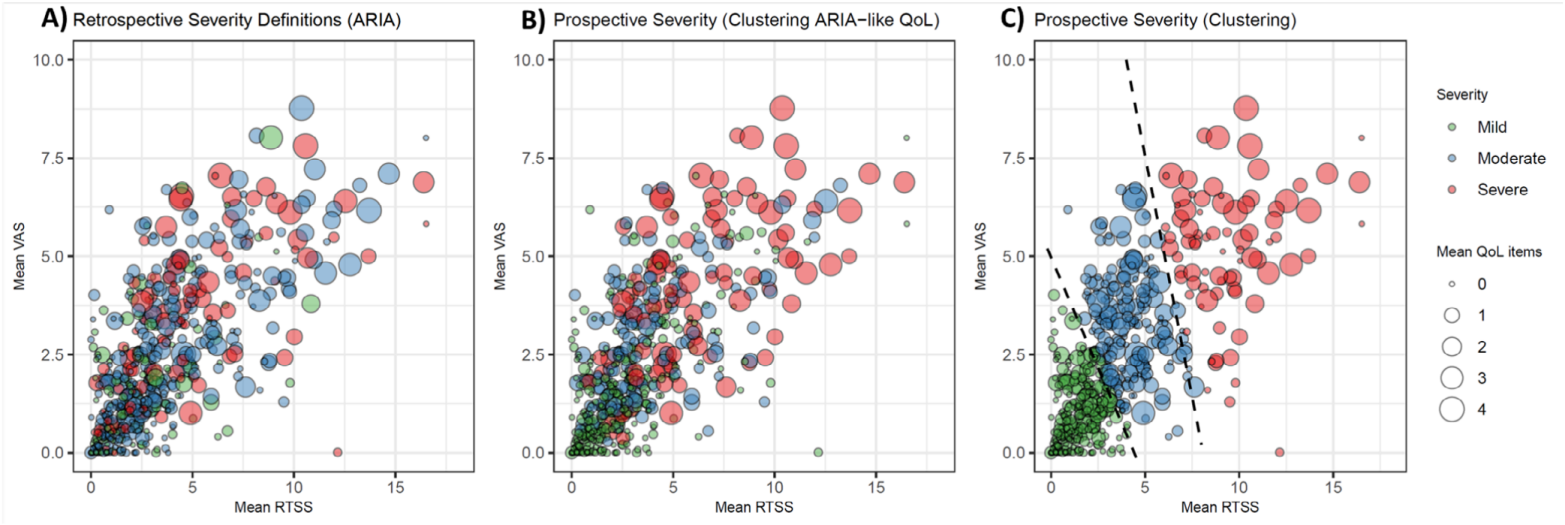
(A) Bubble plot of prospective symptom severity by retrospective ARIA severity definitions (red: Severe, blue: Moderate, green: Mild). Mean balanced accuracy with the clustering from 4C as reference: 0.54. (B) Prospective severity clustering based on unsupervised clustering analysis around medoids (*k* = 3), including only the number of daily affected QoL items. Mean balanced accuracy with the clustering from 4C as reference: 0.62. (C) Prospective severity clustering based on unsupervised clustering analysis around medoids (*k* = 3) including prospective severity of organ‐specific (RTSS), overall (VAS) symptoms, and the number of QoL items affected daily. Sample size in all panels: *n* = 591.

When analysing how well different combinations of the individual severity measures performed on predicting cluster membership, the combination of RTSS plus VAS turned out to have the highest average sensitivity (0.968), specificity (0.979) and balanced accuracy (0.974) with respect to the clustering with all three scores. Interestingly, QoL measures alone ranked the lowest in this analysis with an average balanced accuracy of 0.617 (Table 3).

**TABLE 3:** Clustering comparison (*k* = 3) between all scores (RTSS + VAS + QoL) and all subsets of variables.

Variables included	Average sensitivity	Average specificity	Average balanced accuracy
RTSS + VAS	0.968	0.979	0.974
RTSS	0.845	0.896	0.871
RTSS + QoL	0.843	0.895	0.869
VAS	0.699	0.841	0.770
VAS + QoL	0.688	0.835	0.762
QoL	0.481	0.753	0.617

Given the great performance of RTSS and VAS in predicting the clusters obtained using all three prospective scores, we identified regions in the RTSS–VAS space corresponding to each of the clusters found with the full set of patients (*n* = 591). These are represented by the following inequalities:

- Mild cluster: 2VAS + 2.25RTSS ≤ 9.95
- Moderate cluster: 2VAS + 2.25RTSS > 9.95 & 0.4VAS + 0.98RTSS ≤ 7.92
- Severe cluster: 0.4VAS + 0.98RTSS > 7.92

To assess the applicability of these threshold definitions in different study centres, we re‐performed the clustering exclusively with data from each individual centre. The results in Figure e4 show, that the previously established thresholds matched quite well the clustering results of Marseille, Messina, Porto and Istanbul, while the symptoms of patients in Athens, Izmir, Rome and Tirana would have been underestimated (i.e., often classified as moderate instead of severe). Only in Valencia, symptoms would have been overestimated, due to the fact that only few and overall mildly affected patients were included in this centre.

To assess the generalizability of the findings, we created a bootstrap estimate based on 1000 drawn samples. The clustering based on these samples resulted in ‘lower’ threshold lines for each severity group (Figure e5), leading to the conclusion that the model is sensitive to patients with a very severe phenotype (i.e., high values in both RTSS and VAS). All three clusters showed good stability during the bootstrap analysis (mean Jaccard Index > 0.75) and the cluster containing patients with the lowest mean scores (i.e., the ‘Mild’ cluster) was identified as highly stable (mean Jaccard Index > 0.85) as defined by the authors of the ‘fpc’ package (Table e5).

## 4 Discussion

In a large, longitudinal, multicentre observational study of patients with SAR from culturally and geographically diverse settings, we found that: (i) a retrospective evaluation of SAR severity with the ARIA questionnaire lacks precision in measuring SAR symptoms severity and frequency; (ii) frequency and severity of SAR symptoms can be measured by an e‐Diary by trained patients in the context of an observational study; (iii) clustering methods may facilitate a novel type of ARIA classification, based on prospective, real‐time e‐Diary data; (iv) this novel approach is applicable in different geographic and cultural settings.

### 4.1 ARIA Classification by Retrospective Questionnaire Is Exposed to Recall Bias

Our results confirm [20] that a retrospective clinical history, even if well performed according to international guidelines [6], can be biased. Patients who reported having SAR symptoms almost every day over 5 or more weeks *during* their pollen season recalled only infrequent symptoms (< 4 days a week) for shorter periods (< 4 weeks) when interviewed *after* the pollen season. Similarly, patients with e‐Diary records of sleep disturbances, lost days at work/school, fastidious symptoms, and/or limitations in daily activities *during* their pollen season failed to recall them at the follow‐up visit. Of note, we also observed a retrospective overestimation of SAR symptoms frequency and severity by patients whose e‐Diary showed infrequent symptoms with no impact on sleep or daily activities. Interestingly, this observation was made across geographically and culturally diverse settings. However, we cannot extrapolate our observation to the other questionnaires used to retrospectively classify SAR, such as a physician‐led, 5‐items ARPhyS scale [35], a questionnaire with 17 variables identifying four AR phenotypes in childhood [36], or others [37, 38]. However, considering that the ARIA questionnaire is easy, short, feasible and largely adopted all over the world [6], we speculate that e‐Diaries are more precise also compared to other retrospective SAR questionnaires. Although all patient‐reported outcome measures are subject to different sources of bias, a prospective longitudinal approach may be more accurate, particularly in settings with changing exposure to elicitors [8].

### 4.2 A Novel ARIA Classification, Based on Prospective e‐Diary Recording

Our results also confirm that both frequency and severity of SAR symptoms can be easily measured by an e‐Diary, especially when a high adherence to compilation is supported by a doctor‐patient blended approach [1, 19, 21, 22]. We have established easily a standardised methodology to calculate both symptoms duration and frequency (see Section 2) from prospectively recorded data. Further, our cluster analysis was able to discriminate SAR severity in three classes (mild, moderate, severe), based on the internationally validated parameters and disease severity scores (see Section 2). With this strategy, we could reproduce the six categories of the most recent ARIA classification [23] by combining each frequency class with each of the three severity classes. The criteria used to fix the frequency classes are those used by the ARIA classification [6]. Concerning the three SAR severity classes, we used classification criteria generated by our cluster analysis. Although RTSS and VAS had a higher relevance in these criteria, the four QoL parameters also contributed to our clustering classification. Moreover, our sensitivity analysis discloses enough robustness in different clinical centres.

### 4.3 Clinical Implications: Improving AIT Prescription and Efficacy Awareness

Our study suggests that a prospective ARIA classification may be more precise and improve patient assessment, providing a better knowledge on disease severity and frequency that could be used to improve the selection of patients for AIT in the future [5]. Moreover, a careful evaluation of AIT candidates with an e‐Diary, by establishing a baseline evaluation of SAR severity, would allow a precise measure of the improvement produced by AIT [5] in the following pollen season. Last, even the composition of AIT would be more precise, especially in poly‐sensitised patients living in temperate climates, thanks to a better definition of the temporal and causal effect association between airborne pollen exposure and allergic symptoms [39, 40]. Overall, the use of e‐Diaries in the diagnosis and classification of SAR patients before AIT is started may provide an extraordinary basis for a better AIT prescription, monitoring, and perhaps efficacy. Our study suggests that these hypotheses deserve to be promptly investigated.

### 4.4 Strengths and Limitations

Important strengths of our study are the large number of patients examined with a standardised methodological approach in geographically and culturally diverse regions of Southern European countries. However, some limitations need to be considered. Firstly, the study population is not representative of the general populations in the respective countries. However, they depict a cross‐section of patients suffering from SAR who attend specialised allergy centres. Second, our conclusions need to be replicated in different geographic areas, especially those (e.g., Central and Northern Europe) characterised by a simpler aerobiological and sensitization scenario and among patients suffering from allergic rhinitis and not sensitised to pollen, but mites or other indoor or perennial allergens. Third, e‐Diary data may have their own biases and not always represent the truth. For example, we assume that missing data is missing at random and that missing data in the e‐Diary are more likely to be days with low to no symptoms. Moreover, we have chosen not to consider daily medication in this study, as medication is not considered in ARIA classification. Future studies will have to address the question whether clinical history and retrospective reporting is precise or affected by recall bias when drug assumption is considered. Fourth, our data have been obtained in the context of an observational study, and conclusions on patients' compliance and subjective severity rating, feasibility and reliability of a prospective approach may be different in a real‐life context.

## 5 Conclusions

We propose here a novel and prospective approach to SAR classification, strongly inspired by the current, retrospective ARIA classification. Although more precise, a prospective ‘ARIA’ classification of patients with SAR requires time and effort by both patients and their allergists. Our study suggests the daily electronic diary is much more accurate in reflecting the patient's actual perception of symptoms than retrospective clinical history, which is flawed by recall bias. Future validation studies in real‐life settings will dictate whether prospective e‐Diary will add value to the diagnostic work‐up to define SAR severity and to take the consequent treatment decisions, namely on allergen immunotherapy.

## Funding

S.A. was supported by the EAACI Fellowship Award of the European Academy of Allergy and Clinical Immunology. P.M.M. is funded by the Deutsche Forschungsgemeinschaft (DFG) (MA47/2‐1). The study has been supported by an unrestricted grant from Euroimmun. The Informatics Platform AllergyCARD and the app AllergyMonitor have been kindly provided by TPS Software Production.

## Conflicts of Interest

Stephanie Dramburg reports fees from Allergy Therapeutics, DBV Technologies, Allergopharma, OMRON Healthcare, Stallergenes, Siemens Healthineers, Chiesi, participation in an advisory and data safety board for Allergy Therapeutics, and research funding from Thermo Fisher Scientific. Salvatore Tripodi and Simone Pelosi are co‐founder of TPS Software Production. Salvatore Tripodi reports funds from TPS Production. Simone Pelosi reports personal fees from TPS Software Production. Stefania Arasi reports honoraria from Ulrich, Abbott, DBV, funds from Stallergenes Greer, participation on boards of Novartis, Aimmune and WAO. Paraskevi Xepapadaki reports consulting fees from Novartis and honoraria from Galenica, Glaxo Smith Kline, Menarini, Novartis, Uriach, Nestle, Nutricia. Lucia Caminiti reports participation on board of Aimmune. Cansin Sackesen reports honoraria from UCB. Ozlem Goksel reports honoraria from UCB. Psarros Fotios reports honoraria from Takeda, Abbvie, CSL Behring, GSK, Astra Zeneca, participation board of Takeda. Ulas Uguz reports honoraria from UCB. Ana Margarida Pereira reports funds from Roxall group. João Fonseca reports funding and participation on board of Alerimune, Lda Porto. Laurie Pahus reports honoraria from Glaxo Smith Kline, Astra Zeneca, funds and participation on boards of from Glaxo Smith Kline, Astra Zeneca, Chiesi. Luis Delgado reports funds and honoraria from Thermo Fisher Diagnostics, Leti Pharma, Alerimune, Lda Porto, participation on boards of Laboratorios Vitoria SA, Alerimune, Lda Porto. Mariana Couto reports grants from Roche, funds from EAACI. Paolo M. Matricardi reports research grants from Hycor, EUROIMMUN, and TFS; support for research from Hycor, TFS, MADX and TPS Software Production, and speaker's fees from Hycor, TFS, and MADX outside the present work. All other authors report no conflicts of interest.

## Supporting information


**Figure e1.** Prospectively recorded symptom severity measured by (A) VAS: visual analogue scale and (B) quality of life data by retrospectively determined severity class (ARIA) and study centre; Mean population data plus 95% confidence interval are shown. Note: Plots are only shown for dates in which ≥ 3 patients reported the respective symptom severity. Details on group sizes are shown in Table 1. ATH: Athens (*n* = 77), IST: Istanbul (*n* = 53), IZM: Izmir (*n* = 73), MAR: Marseille (*n* = 71), MES: Messina (*n* = 49), POR: Portugal (*n* = 76), ROM: Rome (*n* = 79), TIR: Tirana (*n* = 86), VAL: Valencia (*n* = 26).
**Figure e2.** Per patient mean score and standard error of prospectively recorded symptom severity (e‐Diary) by study center. RTS: Rhinoconjunctivitis Total Symptom Score (0–18), VAS: Visual Analogue Scale (0–10) of overall symptoms, QOL_items: sum of sleep impairment, work/school productivity impairment, affectation of leisure activities, and perception of troublesome symptoms (0–4). ATH: Athens (*n* = 77), IST: Istanbul (*n* = 53), IZM: Izmir (*n* = 73), MAR: Marseille (*n* = 71), MES: Messina (*n* = 49), POR: Portugal (*n* = 76), ROM: Rome (*n* = 79), TIR: Tirana (*n* = 86), VAL: Valencia (*n* = 26).
**Figure e3**. Proportion of days with reports of affected quality of life by study centre. From top to bottom: affected daily activities/leisure, symptoms perceived as particularly troublesome, impairment of school/work activity, and sleep impairment. Number of records: ATH *n* = 9.148; IST *n* = 5.292; IZM *n* = 7.200; MAR *n* = 6.450; MES *n* = 4.121; POR *n* = 7.618; ROM *n* = 10.730; TIR *n* = 12.080; VAL *n* = 2.262; Total *n* = 64.901.
**Figure e4**. Estimated severity clusters by study centre (red: severe, blue: moderate, green: mild) Dashed lines indicate the global separating lines between clusters based on the clustering analysis on all patients considering RTSS, VAS, and quality of life items. Sample sizes: ATH *n*_mild_ = 27; *n*_moderate_ = 43; *n*_severe_ = 7; IST *n*_mild_ = 26; *n*_moderate_ = 17; *n*_severe_ = 10; IZM n_mild_ = 24; *n*_moderate_ = 30; *n*_severe_ = 19; MAR *n*_mild_ = 9; *n*_moderate_ = 40; *n*_severe_ = 22; MES *n*_mild_ = 11; *n*_moderate_ = 32; *n*_severe_ = 6; POR *n*_mild_ = 5; *n*_moderate_ = 54; *n*_severe_ = 18; ROM *n*_mild_ = 19; *n*_moderate_ = 56; *n*_severe_ = 4; TIR *n*_mild_ = 16; *n*_moderate_ = 46; *n*_severe_ = 24; VAL *n*_mild_ = 5; *n*_moderate_ = 20; *n*_severe_ = 1.
**Figure e5**. Bootstrap estimate based on 5000 drawn samples to assess generalizability of the clustering results. The clustering based on these samples resulted in the dashed threshold lines for each severity group. Black lines indicate the thresholds identified via unsupervised clustering of the study cohort data. Sample size: same as for Figure e4.
**Table e1.** Baseline characteristics of patients with complete study visits and at least 10 days of e‐Diary recording versus patients who were excluded due to incomplete (monitoring).
**Table e2**. Cohort descriptive for T1 by disease severity (ARIA at T1); SPT: skin prick test; SD, standard deviation.
**Table e3**. Comparison of ARIA severity and frequency groups at T0 and T1.
**Table e4**. Mean, maximum and high days of prospectively recorded symptom severity (e‐Diary) by retrospective severity class (T1 ARIA classification).
**Table e5**. Bootstrap estimates of cluster stability
**Table e6**. Sensitivity analysis of clustering around medoids. Sensitivity, specificity and balanced accuracy of clustering results with all included patients and high quality patients (considering clustering results with all patients as reference).

## Data Availability

The data that support the findings of this study are available on request from the corresponding author. The data are not publicly available due to privacy or ethical restrictions.
