# Supplementary material for: Classification of Seasonal Allergic Rhinitis Patients by Prospective Patient‐Recorded Symptoms: The @IT.2020 Project
Source: Allergy. 2026 Sep 2;81(11):3972–84. doi: 10.1111/all.70447 (PMC13753689; doi:10.1111/all.70447)
Supplement: Supplementary file 1 [file ALL-81-3972-s001.docx]

**Classification of seasonal allergic rhinitis patients by prospective patient-recorded symptoms: the @IT.2020 project**

**Short title: Classification of SAR by e-Diary**

**ONLINE REPOSITORY**

Dramburg S^1^**^*^**, Hernandez Toro CJ^1,2*^, Grittner U^2^, Potapova E^1^, Travaglini A^3,4^, Tripodi S^5,6^, Arasi S^1,7^, Pelosi S^8^, Monnati F^3,9,10^, Acar Şahin A^11^, Aggelidis X^12^, Barbalace A^13^, Bourgoin A^14^, Bregu B^15^, Brighetti MA^3^, Caeiro E^16,17^, Caglayan Sozmen S^18^, Caminiti L^13^, Charpin D^14^, Couto M^19^, Delgado L^20,21,22^, Di Rienzo Businco A^5^, Dimier C^14^, Dimou MV^23^, Fonseca JA^21,22^, Goksel O^24^, Hernandez D^25^, Hoffmann TM^1^, Jang DT^26^, Kalpaklioglu F^27^, Lame B^15^, Llusar R^26^, Makris M^12^, Mazon A^26^, Mesonjesi E^15^, Nieto A^26^, Öztürk AB^28^, Pahus L^29^, Pajno G^13^, Panasiti I^13^, Papadopoulos NG^23,30^, Pellegrini E^31^, Pereira AM^21,22^, Pereira M^20,21^, Pinar NM^11^, Priftanji A^15^, Psarros F^32^, Sackesen C^33^, Sfika I^5^, Suarez J^34^, Thibaudon M^35^, Uguz U^36^, Verdier V^14^, Villella V^5^, Xepapadaki P^37^, Yazici D^38,39^, and Matricardi PM^1,10,40^^

^*^ both authors contributed equally to the manuscript

^ corresponding author

^1^ Department of Pediatric Respiratory Care, Immunology and Intensive Care Medicine, Charité – Universitätsmedizin Berlin, Corporate Member of Freie Universität Berlin and Humboldt-Universität zu Berlin, Berlin, Germany.

^2^ Institute of Biometry and Clinical Epidemiology, Charité - Universitätsmedizin Berlin, Berlin, Germany
^3^ Department of Biology, Tor Vergata University, Rome, Italy.

^4^ Italian Aerobiology Monitoring Network - Italian Aerobiology Association, Rome, Italy.

^5^ Pediatric Allergy Unit, Sandro Pertini Hospital, Rome, Italy.

^6^ Allergolology Service, Policlinico Casilino, Rome, Italy

^7^ Pediatric Allergology Unit, Department of Pediatric Medicine, Bambino Gesù Children´s Research Hospital (IRCCS), Rome, Italy.

^8^ TPS Production srl, Rome, Italy.

^9^ INFN & Department of Physics, Tor Vergata University of, Rome, Italy.

^10^ Fraunhofer Institute for Translational Medicine and Pharmacology ITMP, Immunology and Allergology, Berlin, Germany.

^11^ Department of Biology, Faculty of Science, Ankara University, Ankara, Turkey.

^12^ Allergy Unit, 2nd Department of Dermatology and Venereology, National and Kapodistrian University of Athens, University Hospital "Attikon", Athens, Greece.
^13^ Department of Pediatrics- Allergy Unit, University of Messina, Messina, Italy.

^14^ Department of Pneumonology and Allergy, La Timone Hospital, APHM, Aix-Marseille University, Marseille, France.

^15^ Department of Allergology and Clinical Immunology. UHC Mother Teresa, Medical University Tirana, Tirana, Albania.
^16^ MED Mediterranean Institute for Agriculture, Environment and Development & CHANGE Global Change and Sustainability Institute, Institute for Advanced Studies and Research, Universidade de Évora, Évora, Portugal.
^17^ Portuguese Society of Allergology and Clinical Immunology, Lisbon, Portugal.
^18^ Medicana International Izmir Hospital, Izmir, Turkey.

^19^ Immunoallergology, Hospital CUF Trindade, Porto, Portugal.

^20^ Basic and Clinical Immunology Unit, Department of Pathology, Faculty of Medicine, University of Porto, Porto, Portugal.

^21^ RISE-Health, Faculty of Medicine of the University of Porto, Porto, Portugal.

^22^ Allergy Unit, Instituto & Hospital CUF Porto, Porto, Portugal.

^23^ Allergy Department, 2nd Pediatric Clinic, Athens General Children's Hospital "P&A Kyriakou", University of Athens, Athens, Greece.

^24^ Department of Pulmonary Medicine, Division of Immunology, Allergy and Asthma. Faculty of Medicine, Ege University, Izmir, Turkey.

^25^ Department of Allergy, Health Research Institute Hospital La Fe, Valencia, Spain.

^26^ Pediatric Allergy and Pneumology Unit, Children's Hospital La Fe; Health Research Institute La Fe, Valencia, Spain.

^27^ Kırıkkale University School of Medicine, Department of Immunology and Allergic Diseases, Turkey

^28^ Department of Pulmonary Medicine, Division of Allergy and Immunology, Arel University, School of Medicine, Istanbul, Turkey.

^29^ Aix Marseille Univ, APHM, INSERM CIC 1409, INSERM U1263, INRA 1260 (C2VN), Marseille, France

^30^ Division of Infection, Immunity & Respiratory Medicine, Royal Manchester Children's Hospital, University of Manchester, Manchester, UK.

^31^ ARPA- Regional Agency for Environmental Protection, Department of Reggio Calabria,
Calabria, Italy

^32^ Allergy Department, Athens Naval Hospital, Athens, Greece.
^33^ Division of Pediatric Allergy, Koç University School of Medicine, Istanbul, Turkey
^34^ Department of Biology of Organisms and Systems, Area of Botany, University of Oviedo, Oviedo, Spain.

^35^ Réseau National de Surveillance Aérobiologique, Brussieu, France.

^36^ Department of Biology, Faculty of Science, Ege University, Izmir, Turkey.
^37^ Allergy Department, 2nd Pediatric Clinic, National and Kapodistrian University of Athens, Athens, Greece.

^38^ Cellular and Molecular Medicine, KUTTAM, Graduate School of Health Sciences, Koç University, Istanbul, Turkey.
^39^ Swiss Institute of Allergy and Asthma Research (SIAF), University of Zurich, Davos, Switzerland

^40^ Institute of Allergology, Charité – Universitätsmedizin Berlin, Corporate Member of Freie Universität Berlin and Humboldt-Universität zu Berlin, Berlin, Germany

**Word count:** 469

**Tables:** 5

**Figures:** 5

**Corresponding author:**

Paolo Maria Matricardi

Institute of Allergology,

Charité – Universitätsmedizin Berlin

Hindenburgdamm 27,

12203 - Berlin, Germany

[paolo.matricardi@charite.de](mailto:paolo.matricardi@charite.de)

Orchid ID: 0000-0001-5485-0324

**Methods**

**Calculation of Standardized Mean Difference**

The SMD is Cohen’s d for continuous normal distributed data and was calculated for binary and categorical data as proposed by Austin^1^ and Yang and Dalton^2^respectively.

**Statistics of prospectively recorded symptom data**

The scores recorded prospectively were interpolated (RTSS & VAS linear interpolation, QoL items Last Observation Carried Forward) for gaps of up to 3 consecutive days. Population level trajectories of each score were aggregated by centre to obtain the mean daily score and 95% confidence interval in days in which at least 3 patients reported symptoms. Mean daily score trajectories were smoothed using a 7-day rolling window average.

Score summary statistics (mean, maximum, and proportion of highly symptomatic days and proportion of days with reports of an affected QoL item) were calculated for each patient only considering days within the relevant fragmented season(s). Additional comparisons of mean daily scores were performed, only considering patients sensitized to the most commonly reported allergenic source in each centre. The score aggregation was performed only using the days belonging to the relevant fragmented season(s) for each patient.

Highly symptomatic days were arbitrarily considered as:

a) for RTSS and VAS: a daily score higher or equal to the third Quartile of all reports for each score (RTSS >= 5, VAS >= 4);
b) for the number of quality-of-life items: a day with two or more QoL items reported.

The association between the number of days in which patients reported disturbances in each QoL items present on the e-Diary and the T1 ARIA severity of disease (Mild, Moderate, or Severe) was evaluated for each QoL item (affected daily activities/leisure, symptoms perceived as particularly troublesome, impairment of school/work activity, and sleep impairment) with a Poisson regression, with the total number of days with reports from each patient as offset to account for varying follow-up durations and a random intercept by Study Center. Incidence Rate Ratios (IRRs) were estimated for each QoL item considering the Mild ARIA severity group at T1 as reference.

The ARIA classification of disease frequency was applied to the prospective RTSS of patients who recorded symptoms for at least 28 days. Prospective and retrospective ARIA frequency classification was compared via contingency table. Average balanced accuracy, sensitivity, and specificity were estimated considering the prospective classification as reference and the persistent class as the positive class.

**Sensitivity analysis**

Four score quality measures were estimated with each score reported by the patients: Intra-variation Index, Individual Variation Range, Proportion of postponed reports, and Adherence during reporting period between the 5th and the 95th percentile of days^26^. These were used to assess the quality of the data reported by the patients. As sensitivity analysis, the clustering (k=3) with all patients was repeated with the subset of patients that met the following criteria: VAS and RTSS with intra-variation Index higher than 25% of the patients or VAS and RTSS with Individual Variation Range higher than 25% of the patients, and a proportion of postponed recording lower than 25% of the patients and an Adherence to reporting higher than 25% of the patients (n=287 patients). The results of both clustering analyses were compared via a contingency table **[Table e6]**.

References

1. Austin PC. Using the Standardized Difference to Compare the Prevalence of a Binary Variable Between Two Groups in Observational Research. Commun Stat-Simul C. 2009;38(6):1228-1234.
2. Yang D, Dalton JE. A unified approach to measuring the effect size between two groups using SAS. 2012. (2020)
3. Dramburg S, Perna S, Di Fraia M, et al. Validation Parameters of Patient-Generated Data for Digitally Recorded Allergic Rhinitis Symptom and Medication Scores in the @IT.2020 Project: Exploratory Study. JMIR Mhealth Uhealth. 2022;10(6):e31491. doi: 10.2196/31491.

**Legends to the Figures:**

**Figure e1 –** Prospectively recorded symptom severity measured by (A) VAS: visual analogue scale and (B) quality of life data by retrospectively determined severity class (ARIA) and study center; Mean population data plus 95% confidence interval are shown. Note: plots are only shown for dates in which ≥3 patients reported the respective symptom severity. Details on group sizes are shown in Table 1. ATH: Athens (n= 77) , IST: Istanbul (n=53), IZM: Izmir (n= 73) , MAR: Marseille (n=71) , MES: Messina (n= 49) , POR: Portugal (n= 76) , ROM: Rome (n=79) , TIR: Tirana (n=86) , VAL: Valencia (n= 26) .

**Figure e2 -** Smoothed mean of prospectively recorded symptom A) RTSS: rhinoconjunctivitis total symptom score, B) VAS: visual analogue scale and C) quality of life data by retrospectively determined severity class (ARIA at T1) and study center; ATH: Athens (n= 77) , IST: Istanbul (n=53), IZM: Izmir (n= 73) , MAR: Marseille (n=71) , MES: Messina (n= 49) , POR: Portugal (n= 76) , ROM: Rome (n=79) , TIR: Tirana (n=86) , VAL: Valencia (n= 26) .

**Figure e2 -** Per patient mean score and standard error of prospectively recorded symptom severity (e-Diary) by study center. RTS: Rhinoconjunctivitis Total Symptom Score (0-18), VAS: Visual Analogue Scale (0-10) of overall symptoms, QOL_items: sum of sleep impairment, work/school productivity impairment, affectation of leisure activities, and perception of troublesome symptoms (0-4).ATH: Athens (n= 77) , IST: Istanbul (n=53), IZM: Izmir (n= 73) , MAR: Marseille (n=71) , MES: Messina (n= 49) , POR: Portugal (n= 76) , ROM: Rome (n=79) , TIR: Tirana (n=86) , VAL: Valencia (n= 26) .

**Figure e3 -** Proportion of days with reports of affected quality of life by study center. From top to bottom: affected daily activities/leisure, symptoms perceived as particularly troublesome, impairment of school/work activity, and sleep impairment. Number of records: ATH n=9.148; IST n=5.292; IZM n=7.200; MAR n=6.450; MES n=4.121; POR n=7.618; ROM n=10.730; TIR n=12.080; VAL n=2.262; Total n=64.901

**Figure e4 –** Estimated severity clusters by study center (red: severe, blue: moderate, green: mild) Dashed lines indicate the global separating lines between clusters based on the clustering analysis on all patients considering RTSS, VAS, and quality of life items. Sample sizes: ATH n_mild_=27; n_moderate_=43; n_severe_=7; IST n_mild_=26; n_moderate_=17; n_severe_=10; IZM n_mild_=24; n_moderate_=30; n_severe_=19; MAR n_mild_=9; n_moderate_=40; n_severe_=22; MES n_mild_=11; n_moderate_=32; n_severe_=6; POR n_mild_=5; n_moderate_=54; n_severe_=18; ROM n_mild_=19; n_moderate_=56; n_severe_=4; TIR n_mild_=16; n_moderate_=46; n_severe_=24; VAL n_mild_=5; n_moderate_=20; n_severe_=1.

**Figure e5 -** Bootstrap estimate based on 5000 drawn samples to assess generalizability of the clustering results. The clustering based on these samples resulted in the dashed threshold lines for each severity group. Black lines indicate the thresholds identified via unsupervised clustering of the study cohort data. Sample size: same as for Figure e4.

**Figure e1:**

A) **B)**

**Figure e2 –**

**Figure e3 –**

**Figure e4 –**

**Figure e5**

**–**

**Table e1 –** Baseline characteristics of patients with complete study visits and at least 10 days of e-Diary recording versus patients who were excluded due to incomplete (monitoring).

data

**Table e2 -** Cohort descriptives for T1 by disease severity (ARIA at T1); SPT: skin prick test; SD: standard deviation

**Table e3 –** Comparison of ARIA severity and frequency groups at T0 and T1

**Table e4 -** Mean, maximum and high days of prospectively recorded symptom severity (e-Diary) by retrospective severity class (T1 ARIA classification). RTSS = Rhinoconjunctivitis Total Symptom Score, VAS = Visual Analogue Scale of overall symptoms, QOL_items = sum of sleep impairment, work/school productivity impairment, affection of leisure activities and perception of troublesome symptoms. Data is shown as population mean and per-patient averages/median for Max and proportion of highly symptomatic days.

**Table e5 –** Bootstrap estimates of cluster stability

Table e6: Sensitivity analysis of clustering around medoids. Sensitivity, specificity and balanced accuracy of clustering results with all included patients and high quality patients (considering clustering results with all patients as reference)
